# Sex-differences in endotoxemia and trimethylamine N-oxide according to the diet and type 2 diabetes status in coronary heart disease patients: from the CORDIOPREV study

**DOI:** 10.3389/fcvm.2025.1527406

**Published:** 2025-10-21

**Authors:** Helena Garcia-Fernandez, Javier Arenas-Montes, Francisco M. Gutierrez-Mariscal, Juan F. Alcala-Diaz, Alisa Allais, Antonio P. Arenas-de Larriva, Diego Luque-Cordoba, Jose D. Torres-Peña, Juan L. Romero-Cabrera, Raul M. Luque, Feliciano Priego-Capote, Javier Delgado-Lista, Jose Lopez-Miranda, Antonio Camargo

**Affiliations:** ^1^ Lipids and Atherosclerosis Unit, Department of Internal Medicine, Reina Sofía University Hospital, Cordoba, Spain; ^2^ Department of Medical and Surgical Sciences, University of Cordoba, Cordoba, Spain; ^3^ Maimonides Institute for Biomedical Research in Cordoba (IMIBIC), Cordoba, Spain; ^4^ CIBER Fisiopatologia Obesidad y Nutricion (CIBEROBN), Instituto de Salud Carlos III, Madrid, Spain; ^5^ Department for Sustainable Development and Ecological Transition, Unit Biology: Food Health and Environment, Università Piemonte Orientale, Vercelli, Italy; ^6^ Department of Analytical Chemistry and Nanochemistry University Institute, University of Cordoba, Cordoba, Spain; ^7^ CIBER de Fragilidad y Envejecimiento Saludable (CIBERFES), Instituto de Salud Carlos III, Madrid, Spain; ^8^ Department of Cell Biology, Physiology, and Immunology, University of Cordoba, Cordoba, Spain

**Keywords:** cardiovascular diseases, type 2 diabetes mellitus, sexual dimorphism, endotoxemia, TMAO, CORDIOPREV

## Abstract

**Background:**

Cardiovascular disease (CVD) develops in men earlier in life but CVD women have a higher risk of cardiovascular mortality than CVD men. In addition, co-occurrence with type 2 diabetes mellitus (T2DM) increases CVD risk. We aimed to evaluate sex differences in endotoxemia and trimethylamine N-oxide (TMAO) plasma levels, in co-occurrence with T2DM in coronary heart disease (CHD) patients, and their potential sex-specific modulation by the consumption of healthy diets.

**Methods:**

This study was carried out within the framework of the CORDIOPREV study, a clinical trial which included 1,002 (827 men and 175 women) with CHD, of whom 462 had no T2DM, 350 had T2DM, and 190 were newly diagnosed with T2DM at recruitment. Plasma lipopolysaccharide (LPS) was measured by LAL colorimetric assay and TMAO by HPLC. Intima-media thickness of both common carotid arteries (IMT-CC) and carotid plaques were assessed ultrasonically.

**Results:**

LPS and TMAO plasma levels were lower in CHD non-T2DM women than CHD non-T2DM men (both, *P*-value <0.05), whereas no sex differences were observed in CHD T2DM patients, or CHD newly-diagnosed T2DM patients. These sex differences were consistent with lower IMT-CC and a smaller number of plaques in CHD non-T2DM women than in CHD non-T2DM men, with no sex differences found in CHD T2DM patients, or CHD newly-diagnosed T2DM patients. In contrast, C-reactive protein plasma levels were higher in CHD T2DM women than CHD T2DM men (*P* = 0.012 and *P* = 0.001, respectively), with no sex differences found in CHD non-T2DM patients. Both, LF and Med diets reduced the LPS plasma levels in CHD men and women newly diagnosed with T2DM.

**Conclusions:**

Our results suggest that the sex differences in CHD patients are influenced by the presence of T2DM. Moreover, our results also suggest that the cardiovascular risk associated to T2DM, in co-occurrence with CVD, is higher in women than in men.

## 1 Introduction

CVD develop at an after age for women compared to men, but age-adjusted analyses have shown that women displayed higher CVD rates between 55 and 75 years old (1). Although women overall have a lower prevalence of CVD than men (2), once women have developed CVD, they have a higher risk of cardiovascular mortality than men (3). In the last decades, considerable evidence has been reported on sex-related differences in terms of genetic, molecular mechanism, prevalence and severity of coronary heart disease (CHD) (4–6). In line with this, the atherosclerotic plaque rupture or erosion and arterial thrombus formation also shows sex-specific differences. Indeed, it is well established that plaque rupture is more common in men, whereas plaque erosion is more prevalent in women (7–9).

The incidence of metabolic diseases and their co-morbidities is also sexually dimorphic (10, 11). For example, the prevalence of metabolic syndrome (MetS), a cluster of characteristics associated with an increased risk of type 2 diabetes mellitus (T2DM) and CVD (12), differs according to age, ethnicity, sex, diet and levels of physical activity (13, 14). This syndrome is conventionally diagnosed when a threshold of 3 of 5 criteria is reached; yet, 10 different combinations of such criteria are in fact possible, each with a different pathophysiology. In a similar way, the prevalence of each MetS risk factor also differs by sex and country (14, 15). In addition, increasing evidence points towards certain biological mechanisms associated with T2DM that independently increase the risk of CVD in diabetic patients. Indeed, T2DM incidence has been associated to a higher risk of CVD, and has been estimated at 2–4 times that of the nondiabetic population (16).

Circulating lipopolysaccharide (LPS) contributes to low-grade chronic inflammation, which favors the development of atherosclerosis, the pathogenic substrate of CVD (17). It is therefore plausible that differences in intestinal barrier integrity, and the subsequent absorption of bacterial components such as LPS, may contribute to the sexual dimorphism in CVD. Moreover, an association has been shown between endotoxemia and the risk of developing diabetes (18), which also increases the risk of CVD (16).

Sex seems to be an important factor in the synthesis of TMAO, and sex differences in the plasma levels of this metabolite may help to account, at least partially, for the differences in the incidence of CVD according to sex. In this context, experiments in animal models have shown that the expression and activity of hepatic flavin monooxygenases 3, the isoform that preferentially transforms TMA into the pro-atherogenic TMAO, together with TMAO plasma levels, are significantly higher in female mice than in males (19, 20).

Based on this previous evidence, we explored the sex differences of endotoxemia and TMAO plasma levels, in addition to the potential influence of the prevalence of T2DM in the cardiovascular risk assessed by arterial injury and carotid plaques according to sex in CHD patients. Indeed, it has been previously described that the co-occurrence of CHD with T2DM markedly increases the risk of macrovascular complication and mortality (21, 22). Thus, we aimed to evaluate sex differences in endotoxemia and TMAO plasma levels, in addition to the cardiovascular risk assessed by arterial injury and carotid plaques, in co-occurrence with T2DM in CHD patients, and their potential sex-specific modulation by the consumption of the healthy diets, low-fat and Mediterranean (Med), in the framework of the CORDIOPREV study.

## 2 Methods

### 2.1 Study participants

The current work was conducted within framework of the CORDIOPREV study (https://www.Clinical Trials.gov Identifier: NCT00924937), an ongoing prospective, randomized, open, controlled trial in 1,002 patients with CHD who had their last coronary event over six months before enrolling, and who followed two different dietary models (a low-fat diet and the Mediterranean diet) over a period of seven years, in addition to conventional treatment for CHD. CORDIOPREV inclusion and exclusion criteria can be summarized as follows: patients were eligible if they were over 20 years old but under 75, had established CHD without clinical events in the last 6 months, were thought to be capable of following a long-term dietary intervention, and did not have severe diseases or an estimated life expectancy of less than seven years (23). At the beginning of the CORDIOPREV study, 350 patients out of 1,002 had T2DM (diab group, 280 men and 70 women). In the patients without established diabetes, an oral glucose tolerance test was performed during recruitment. T2DM was defined by levels of HbA1c <6.5%, fasting plasma glucose <126 mg/dl and 2 h plasma glucose in the 75 gr OGTT <200 mg/dl, according to the American Diabetes Association (ADA) diagnosis criteria (24). The results confirmed that 462 patients out of 1,002 did not have diabetes (non-diab group, 389 men and 73 women); however, 190 out of 1,002 were newly-diagnosed patients who had been diagnosed with short-duration diabetes and were not receiving glucose-lowering treatment (new-diab group, 158 men and 32 women). We followed the criteria of the Third Report of the National Cholesterol Education Program (NCEP) Expert Panel on Detection, Evaluation and Treatment of High Blood Cholesterol in Adults (Adult Treatment Panel III) to assess the presence of MetS (25). The CORDIOPREV study has been approved by the Reina Sofia (Cordoba) University Hospital Ethics and Research Committees. All the participants agreed to their inclusion in these studies. The trial protocol and all its amendments were approved by the Reina Sofia University Hospital Clinical Research Ethics Committee, following the Helsinki Declaration and good clinical practice.

### 2.2 Study design

The study design has been previously described (23). Briefly, participants were randomized to receive two diets: a Mediterranean (Med) diet or a low-fat (LF) diet. The LF diet consisted of <30% total fat (<10% saturated fat, 12%-14% MUFA fat, and 6%–8% PUFA fat), 15% protein and a minimum of 55% carbohydrates. The Med diet comprised a minimum of 35% of calories as fat (22% MUFA fat, 6% PUFA fat and <10% saturated fat), 15% proteins and a maximum of 50% carbohydrates. In both diets, the cholesterol content was adjusted to <300 mg/d. The outcomes are ascertained on a yearly basis by a Clinical Events Committee whose members are blinded to the intervention group. During the programmed visits, patient compliance is checked based on the scores of adherence to the Med and LF dietary patterns and by measuring selected biological plasma variables, such as the fatty acids profile. Dietary adherence was assessed with the 14-point Mediterranean Diet Adherence Screener and 9-point low-fat diet adherence (26). No energy restriction was implemented and the study team explicitly did not promote physical activity. The dietary intervention in the CORDIOPREV study included individual face-to-face visits every 6 months, group sessions every 3 months, and telephone calls every 2 months, all of which aimed at guaranteeing frequent contact between the patients and dietitians (at least 12 interactions per year). During the study, the following interventions were done: regular contacts, group sessions, monitoring of adherence, goal setting, social support, and the provision of foods.

### 2.3 Clinical plasma parameters

Blood was collected in tubes containing EDTA to give a final concentration of 0.1% EDTA. The plasma was separated from the red blood cells by centrifugation at 1,500 × g for 15 min at 4°C. Analytes in the frozen samples, blinded to the team members, were analyzed centrally by members of the laboratory research team at the Lipid and Atherosclerosis Unit at Reina Sofia University Hospital. The clinical plasma parameters were measured as previously described (27). Briefly, the different parameters were measured using spectrophotometric techniques (enzymatic colorimetric methods): hexokinase method for glucose, chemiluminescence for insulin, glycated haemoglobin by high performance liquid chromatography and oxidation-peroxidation for total cholesterol, high-density lipoprotein cholesterol (HDL-C) and triglycerides, immunoturbidimetry for C-reactive protein (28, 29). The low-density lipoprotein cholesterol (LDL-C) was calculated using the Friedewald formula (provided the triglyceride level was <300 mg/dl) (30).

### 2.4 Measurement of LPS

The liposaccharide (LPS) was measured using the limulus amebocyte lysate test (QCL-1000 Chromogenic LAL (LonzaIberica S.A., Spain), as previously (31). LPS binding protein (LBP) levels were determined using a human LBP ELISA kit (HycultBiotech, Netherlands).

### 2.5 Measurement of TMAO in plasma

TMAO plasma levels were measured using a 1,200 series LC system from Agilent Technologies coupled with an Agilent 6,460 triple quadrupole mass spectrometer from Agilent Technologies (Palo Alto, CA, USA). The MRM transitions used in this research are listed in Supplementary Table S1. The Agilent MassHunter Workstation software (version B.03.01, Agilent Technologies, Santa Clara, CA, USA) was used for data acquisition, and Agilent MassHunter Workstation Quantitative Analysis software was used for the quantitative analysis (version B.07.00).

### 2.6 Carotid ultrasonography

We assessed the cardiovascular risk was assessed the intima-media thickness intima-media thickness of both common carotid arteries (IMT-CC), considered an early marker of atherosclerosis (32), and more recently an indicator of arterial injury. Moreover, the carotid study also included the determination of the number and height carotid plaques. This analysis was performed by technicians blinded to the clinical information and previous imaging. The carotid study was ultrasonically assessed bilaterally by quantification of intima-media thickness of both common carotid arteries (IMT-CC) and carotid plaques (number and height) as previously (33).

### 2.7 Statistical analysis

**Figure 1:**
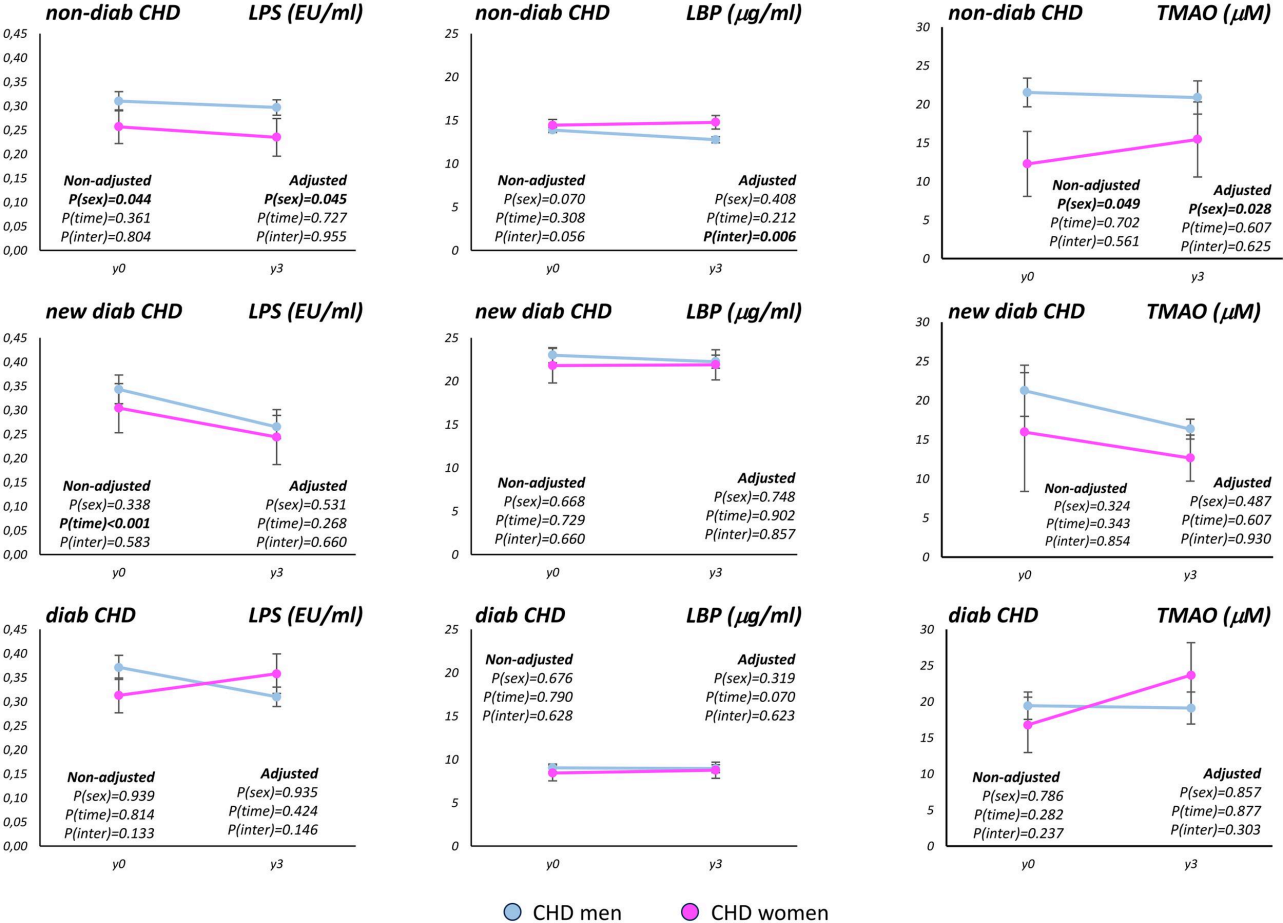
Baseline LPS, LBP and TMAO plasma levels and changes after 3 years of the consumption of healthy diets according to sex in each of the T2DM group. LPS: plasma levels of lipopolysaccharide; LBP, plasma levels of lipopolysaccharide binding protein; TMAO, plasma levels of trimethylamine N-oxide; T2DM, type 2 diabetes mellitus; CHD, coronary heart disease. The determinations were carried out at baseline (y0) and after 3 years of dietary intervention (y3), which consisted in the consumption of a low-fat or a Mediterranean diet. non-diab, CHD patients without type 2 diabetes mellitus; new-diab, CHD patients with recently diagnosed type 2 diabetes mellitus, and without treatment for diabetes; diab, CHD patients with type 2 diabetes mellitus, and under treatment for diabetes. Baseline values and values after 3 years of dietary intervention were analyzed together by ANOVA for repeated measures in order to test sex-specific changes induced by the consumption of healthy diets: the variables for age, body mass index, total cholesterol, low-density lipoproteins, and blood pressure were used as co-variables in the adjusted models. P(sex): *p*-value according to the sex, P(time): *p*-value for time. P(inter): *p*-value for interaction between sex and time.

The PASW statistical software package, version 20.0 (IBM Inc., Chicago, IL, USA), was used for univariate statistical analyses of the data. Multiple logistic regression analysis was carried out including the baseline values for age, BMI, waist circumference, insulin, total cholesterol (TC), HDL-c, LDL-c, triglycerides, C-reactive protein, and blood pressure as dependent variables and T2DM (yes/no) as independent variable. The variables corresponding to plasma levels of glucose and HbA1c were removed from the multiple logistic regression analysis as these variables are diagnostic criteria for T2DM. We used One-way ANOVA to test the differences between groups at baseline. The chi-square test was applied to establish differences in T2DM and MetS prevalence, and MetS criteria analysis. Baseline values and values after 3 years of dietary intervention were analyzed together by ANOVA for repeated measures with time as intra-subject factor, and sex as the inter-subject factor, in order to test sex-specific changes induced by the consumption of healthy diets (Figure 1), low-fat diet (Supplementary Figure S2) and Mediterranean diet (Supplementary Figure S3). We used time as intra-subject factor, and diet as the inter-subject factor, in order to test diet-specific changes induced by the consumption of low-fat diet and Mediterranean diet in CHD men (Supplementary Figure S4) and CHD women (Supplementary Figure S5). The variables for age, body mass index, total cholesterol, low-density lipoproteins, and blood pressure were used as co-variables in the adjusted models. The *post hoc* statistical analysis was completed using Bonferroni's comparison test. *P*-values <0.05 were considered statistically significant.

## 3 Results

### 3.1 Baseline characteristics according to sex of the CHD patients

We observed a higher age, BMI, plasma levels of TC, HDL-c, LDL-c, C-reactive protein, and diastolic blood pressure, but lower waist circumference in CHD women than in CHD men (all, *p* < 0.05) (Table 1). These sex differences were found between CHD men and CHD women, independently of the T2DM status.

**Table 1:** Baseline characteristic of the CORDIOPREV population according to sex.

Variable	Sex	all CHD	non-diab CHD	new diab CHD	diab CHD	*P*-value diab
Age (years)	Men	59.1 ± 0.3	57.4 ± 0.5^a^	59.4 ± 0.7^b^	61.3 ± 0.5^b^	<0.001
	Women	61.8 ± 0.6	59.1 ± 1.1^a^	63.1 ± 1.6^a,b^	64.0 ± 0.9^b^	0.002
	*p*-value sex	<0.001	0.150	0.038	0.011	
BMI (kg/m^2^)	Men	31.0 ± 0.1	30.3 ± 0.2^a^	30.8 ± 0.3^a^	32.1 ± 0.3^b^	<0.001
	Women	31.9 ± 0.4	30.5 ± 0.6^a^	32.5 ± 0.8^a,b^	33.1 ± 0.6^b^	0.010
	*p*-value sex	0.018	0.810	0.053	0.074	
Waist circunference (cm)	Men	106.1 ± 0.4	103.7 ± 0.5^a^	105.8 ± 0.9^a^	109.5 ± 0.6^b^	<0.001
	Women	101.0 ± 1.0	96.3 ± 1.5^a^	101.9 ± 1.9^a,b^	105.4 ± 1.7^b^	<0.001
	*p*-value sex	<0.001	<0.001	0.063	0.007	
Fasting Glucose (mg/dl)	Men	112.9 ± 1.2	93.8 ± 0.5^a^	111.0 ± 2.0^b^	140.4 ± 2.7^c^	<0.001
	Women	117.8 ± 3.8	91.2 ± 1.2^a^	107.0 ± 3.3^a^	151.5 ± 7.9^b^	<0.001
	*p*-value sex	0.125	0.048	0.392	0.095	
Fasting Insulin (mU/L)	Men	11.0 ± 0.4	8.9 ± 0.3^a^	11.7 ± 0.9^b^	13.4 ± 0.9^b^	<0.001
	Women	11.2 ± 0.9	8.5 ± 0.6^a^	11.3 ± 1.2^a,b^	14.2 ± 2.0^b^	0.011
	*p*-value sex	0.768	0.581	0.846	0.707	
HbA1c (%)	Men	6.62 ± 0.04	5.89 ± 0.02^a^	6.70 ± 0.07^b^	7.58 ± 0.08^c^	<0.001
	Women	6.80 ± 0.10	5.92 ± 0.04^a^	6.53 ± 0.08^b^	7.84 ± 0.17^c^	<0.001
	*p*-value sex	0.064	0.513	0.293	0.148	
TC (mg/dl)	Men	157.3 ± 1.0	159.7 ± 1.5^a^	162.3 ± 2.5^a^	151.1 ± 1.7^b^	<0.001
	Women	167.1 ± 2.7	171.6 ± 4.4^a,b^	177.5 ± 6.3^a^	157.7 ± 3.8^b^	0.013
	*p*-value sex	<0.001	0.003	0.015	0.090	
HDL-c (mg/dl)	Men	41.1 ± 0.3	43.1 ± 0.5^a^	40.6 ± 0.8^b^	38.7 ± 0.5^b^	<0.001
	Women	47.3 ± 1.0	51.1 ± 1.4^a^	47.6 ± 1.9^a,b^	43.2 ± 1.5^b^	<0.001
	*p*-value sex	<0.001	<0.001	<0.001	<0.001	
LDL-c (mg/dl)	Men	87.8 ± 0.9	90.7 ± 1.2^a^	90.9 ± 2.1^a^	82.0 ± 1.6^b^	<0.001
	Women	92.3 ± 2.1	96.6 ± 3.6^a^	99.8 ± 4.5^a^	84.5 ± 2.9^b^	0.008
	*p*-value sex	0.038	0.068	0.091	0.472	
TAG (mg/dl)	Men	135.8 ± 2.4	123.7 ± 3.2^a^	143.5 ± 5.8^b^	148.2 ± 4.5^b^	<0.001
	Women	133.6 ± 5.4	116.0 ± 7.4^a^	135.6 ± 12.7^a,b^	151.1 ± 9.0^b^	0.012
	*p*-value sex	0.706	0.338	0.576	0.772	
CRP (mg/L)	Men	2.34 ± 0.07	2.02 ± 0.10^a^	2.62 ± 0.17^b^	2.63 ± 0.13^b^	<0.001
	Women	2.99 ± 0.18	2.43 ± 0.27^a^	3.15 ± 0.39^a,b^	3.51 ± 0.28^b^	0.021
	*p*-value sex	<0.001	0.100	0.194	0.003	
Systolic BP (mm Hg)	Men	138.2 ± 0.7	136.1 ± 1.0^a^	136.6 ± 1.6^a^	142.0 ± 1.2^b^	<0.001
	Women	141.4 ± 1.6	135.8 ± 2.4^a^	140.8 ± 3.4^a,b^	147.6 ± 2.6^b^	0.004
	*p*-value sex	0.055	0.902	0.281	0.042	
Diastolic BP (mm Hg)	Men	77.6 ± 0.4	78.5 ± 0.6^a^	77.4 ± 0.9^a^	76.6 ± 0.6^a^	0.073
	Women	75.3 ± 0.8	76.6 ± 1.1	74.4 ± 1.9	74.4 ± 1.3	0.392
	*p*-value sex	0.011	0.160	0.185	0.134	

Means values ± S.E.M. BMI: body mass index; HbA1c, glycated hemoglobin A1c; TC, total cholesterol; HDL, high-density lipoprotein cholesterol; LDL-c, low-density lipoprotein cholesterol; TAG, triacylglycerides; CRP, C-reactive protein; BP, blood pressure; CHD, coronary heart disease; non-diab, CHD patients without type 2 diabetes mellitus, new-diab, CHD patients with recently diagnosed type 2 diabetes mellitus, and without treatment for diabetes, diab, CHD patients with type 2 diabetes mellitus, and under treatment for diabetes; *P*-value diab, One-way ANOVA *p*-value between non-diab CHD, new diab CHD and diab CHD groups in men or women separately. Values with different letters are statistically significant different in Bonferroni *post hoc* multiple comparison tests; *P*-value sex, One-way ANOVA *p*-value between all CHD men and all CHD women, or between men and women in non-diab CHD, new diab CHD and diab CHD groups separately.

### 3.2 Baseline characteristics according to T2DM status in CHD men and CHD women

We also observed that age, BMI, waist circumference, plasma levels of glucose, insulin, HbA1c, total cholesterol (TC), HDL-c, LDL-c, triglycerides, C-reactive protein, and systolic blood pressure all increased from the non-diab to the new-diab group and to the diab group in both CHD men and CHD women (all, *p* < 0.05) (Table 1). These differences between groups according to their T2DM status were found in CHD men and CHD women separately.

### 3.3 Baseline characteristics according to sex and T2DM in CHD patients

We found statistically significant sex differences in waist circumference, plasma levels of TC, and HDL-c, in the non-diab, new-diab and diab groups separately (Table 1). However, sex-differences in age, C-reactive protein, and systolic blood pressure were only observed between CHD diab men and CHD diab women. In fact, age, C-reactive protein, and systolic blood pressure were higher in CHD diab women than in CHD diab men (*p* = 0.011, *p* = 0.003, and *p* = 0.042, respectively), whereas no differences were observed between sexes in the non-diab group (Table 1).

### 3.4 Sex-dependent prevalence of T2DM and MetS in CHD patients

We observed that the percentage of women with MetS in the CORDIOPREV population was higher than the percentage of men (*P* = 0.010). Indeed, the percentage of women with a small number of MetS criteria was lower than men, but the percentage of women with a greater number of MetS criteria was higher than men (*P* = 0.037). However, we did not observe statistically differences in the percentage of men and women according to the T2DM status, while a trend for lower percentage of non-diab and higher percentage of diab women than men was noticeable (Table 2). A multiple logistic regression analysis was conducted to assess the relationship of the anthropometric and biochemical variables and the prevalence of T2DM (new-diab and diab groups together vs. non-diab group) (Supplementary Figure S1). Among the MetS criteria (Supplementary Table S2), we found a higher percentage of women following the criteria of waist circumference and HDL plasma levels than men (*P* < 0.001 and *P* = 0.001, respectively), whereas no differences were found in the criteria based on glucose, triacylglycerides and hypertension.

**Table 2:** Sex-dependent prevalence of T2DM in CHD patients.

T2DM groups	Men (%)	Women (%)	*p*-value
non-diab	47.04	41.71	0.287
new-diab	19.11	18.29	
diab	33.86	40.00	
non-diab	47.04	41.71	0.199
new-diab + diab	52.96	58.29	

T2DM, type 2 diabetes mellitus; CHD, coronary heart disease; non-diab, CHD patients without type 2 diabetes mellitus, new-diab, CHD patients with recently diagnosed type 2 diabetes mellitus, and without treatment for diabetes, diab, CHD patients with type 2 diabetes mellitus, and under treatment for diabetes; The chi-square test was applied to establish differences in T2DM prevalence.

### 3.5 Sex-dependent differences in IMT-CC and carotid plaques in CHD patients

We found that IMT-CC and the number of plaques were higher in CHD men than in CHD women (both, *p* < 0.05), whereas no sex differences were found in the percentage of carotid plaques presence and plaques height. The sex differences found between CHD men and CHD women, are independent of the T2DM status.

### 3.6 Differences in IMT-CC and carotid plaques according to T2DM status in CHD men and CHD women

We found that IMT-CC increased from the non-diab to the new-diab group and to the diab group in both, CHD men and CHD women (both, *p* < 0.001). We observed that the number of plaques and the plaques height increased from the non-diab to the new-diab group and to the diab group in CHD men (*p* = 0.039 and *p* = 0.025, respectively), which was not observed in CHD women. However, a trend for the increase of the number of plaques from the non-diab to the new-diab group and to the diab group in CHD women was noticeable. These differences between groups according to their T2DM status were found in CHD men and CHD women separately. Finally, no differences in the percentage of carotid plaques presence were observed according to T2DM status in CHD men nor CHD women.

### 3.7 Differences in IMT-CC and carotid plaques according to sex and T2DM in CHD patients

We observed that IMT-CC and the number of plaques were lower in CHD non-diab women than in CHD non-diab men, whereas no differences were observed between sexes in CHD new-diab or CHD diab groups (Table 3). Thus, our results showed sex differences in IMT-CC and the number of plaques in non-diab CHD patients, while no sex differences we found in CHD diab patients.

**Table 3:** Sex-dependent differences in IMT-CC and carotid plaques in CHD patients.

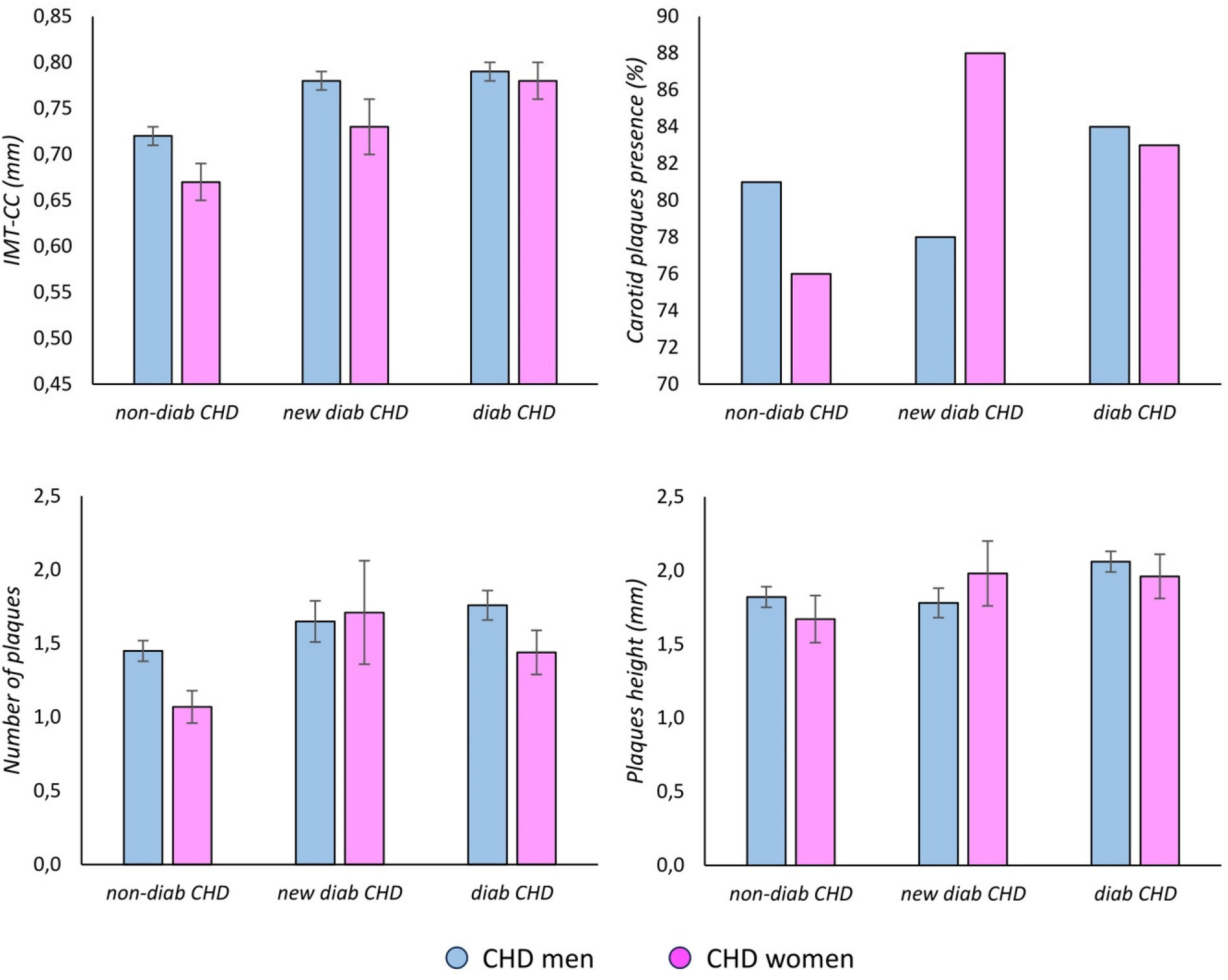						
Variable	Sex	All CHD	non-diab CHD	new diab CHD	diab CHD	*P*-value diab
IMT-CC (mm)	Men	0.75 ± 0.01	0.72 ± 0.01^a^	0.78 ± 0.01^b^	0.79 ± 0.01^b^	<0.001
	Women	0.72 ± 0.01	0.67 ± 0.02^a^	0.73 ± 0.03^a,b^	0.78 ± 0.02^b^	<0.001
	*p*-value sex	0.032	0.013	0.260	0.599	
Carotid plaques presence (%)	Men	82	81	78	84	0.338
	Women	81	76	88	83	0.507
	*p*-value sex	0.865	0.473	0.338	0.776	
Number of plaques	Men	1.61 ± 0.05	1.45 ± 0.07^a^	1.65 ± 0.14^a,b^	1.76 ± 0.10^b^	0.039
	Women	1.33 ± 0.10	1.07 ± 0.11	1.71 ± 0.35	1.44 ± 0.15	0.056
	*p*-value sex	0.037	0.033	0.881	0.133	
Plaques height (mm)	Men	1.90 ± 0.04	1.82 ± 0.07	1.78 ± 0.10	2.06 ± 0.07	0.025
	Women	1.85 ± 0.10	1.67 ± 0.16	1.98 ± 0.22	1.96 ± 0.15	0.347
	*p*-value sex	0.604	0.363	0.486	0.567	

Means values ± S.E.M. or percentage for carotid plaques presence; IMT-CC, intima-media thickness of both common carotid arteries; ApoA, apolipoprotein A; CHD, coronary heart disease; non-diab, CHD patients without type 2 diabetes mellitus, new-diab, CHD patients with recently diagnosed type 2 diabetes mellitus, and without treatment for diabetes, diab, CHD patients with type 2 diabetes mellitus, and under treatment for diabetes; *P*-value diab, One-way ANOVA *p*-value between non-diab CHD, new diab CHD and diab CHD groups in men or women separately. Values with different letters are statistically significant different in Bonferroni *post hoc* multiple comparison tests; *P*-value sex, One-way ANOVA *p*-value between all CHD men and all CHD women, or between men and women in non-diab CHD, new diab CHD and diab CHD groups separately. Carotid plaques presence statistical differences were analyzed by the chi-square test.

### 3.8 Endotoxemia and TMAO modulation by the consumption of healthy diets in CHD patients

We explored the modulation of the LPS, LBP and TMAO plasma levels by the consumption of a LF and the Med diet. These analyses were carried out taken together both LF and Med diets and separately for LF and Med diets. The consumption of healthy diets administered in our study for 3 years reduced the LPS plasma levels in new-diab group (*p* < 0.001), whereas no diet effect was observed in LPS plasma levels in non-diab and diab groups (Figure 1). We also found a reduction on the LPS plasma levels in new-diab group by the consumption of the LF and by the consumption of the Med diet (Supplementary Figures S2, S3, respectively). In addition, we observed lower LPS plasma levels in CHD non-diab women than in CHD non-diab men (*P* = 0.044) taken together baseline and y3 plasma levels, whereas no sex differences were found in the CHD new-diab and CHD diab groups (Figure 1). Moreover, we found no modulation by the consumption of healthy diets nor sex differences in the LBP plasma levels. When we analyze the effect of LF and MED diets LPS plasma levels by sex separately (Supplementary Figures S4, S5), we observed a reduction of the LPS plasma levels in new-diab group in men and women irrespective of the diet consumed (*p* < 0.001 and *p* = 0.031, respectively), whereas no diets effect was observed in LPS plasma levels in non-diab and diab groups.

By other hand, we did not observe changes on the TMAO plasma levels by the consumption of LF and Med diets (Figure 1, Supplementary Figures S2, S3). However, we observed lower TMAO plasma levels in CHD non-diab women than in CHD non-diab men (*P* = 0.049) (Figure 1). We found comparable results on TMAO plasma levels when we analyzed LF and Med diets separately (Supplementary Figures S1, S2).

## 4 Discussion

Sex differences are currently focus of researching, and the analyzing of CHD patients according to the sex and the diabetic status may uncover a potential sexual dimorphism in the increase of CVD risk associated to the co-occurrence with T2DM. We explored whether the sexual dimorphism in endotoxemia and TMAO plasma levels in CHD patients is influenced by the presence of diabetes, in addition to its potential modulation by the consumption of the health diets, LF and Med. We found that a reduction on the LPS plasma levels in new-diab group by the consumption of the LF and by the consumption of the Med diet independently of the sex of the patients. No diet effect was observed for TMAO plasma levels. However, plasma levels of LPS and TMAO were lower in CHD non-diab women than CHD non-diab men, whereas no sex differences were observed in the new-diab and diab groups. Thus, sex differences observed in CHD non-diabetic patients were not present in CHD diab patients, while the CHD new-diab patients displayed intermediate behavior. These sex differences were consistent with lower IMT-CC and a smaller number of plaques in CHD non-diab women than CHD non-diab men, with no sex differences in the new-diab group and diab groups. This latter is especially important as the atherosclerotic plaque stability also shows sex-specific differences (7).

LPS is a pro-atherogenic bacterial component which induces inflammation and subsequently IR (34, 35). Although our study did not show differences in LPS plasma levels between the different groups according to T2DM, we observed sex differences in LPS plasma levels in one of the groups of patients according to T2DM. Indeed, LPS plasma levels were lower in CHD non-diab women than CHD non-diab men, whereas no sex differences were found in the new-diab group and diab groups.

Both LBP and CRP are acute phase proteins (36). While LBP responds to invasive bacterial infection and presents LPS to important cell-surface pattern recognition receptors called CD14 and TLR4 (37), CRP binds to phosphocholine expressed on the surface of damaged cells, as well as to the polysaccharides and peptosaccharides present on bacteria, and helps to promote phagocytosis and the innate immune response against foreign infectious pathogens (38, 39). In line with this, the low-grade inflammation linked to high plasma levels of inflammatory molecules such as CRP, have apported evidence for the positive association between elevated CRP levels and T2DM (40, 41). In fact, the multiple logistic regression analysis conducted in our study to assess the relationship of the anthropometric and biochemical variables and T2DM showed to CRP plasma levels as the more strength positively variable.

Our research showed lower LBP plasma levels in diab patients than in new-diab patients, with non-diab patients displaying intermediate levels in both CHD men and CHD women, with no sex differences found. In contrast, CRP plasma levels were different between sexes according to T2DM status. While CRP in plasma levels in CHD men was higher in the new-diab group than the non-diab group, with the diab group showing intermediate levels, no differences were found in CHD women according to T2DM. In addition, we found higher CRP plasma levels in CHD diab women than in CHD diab men, whereas no sex differences in CRP levels were found in the non-diab and new-diab groups. This is especially important if we take into account that CRP levels are an indicator of inflammation, a major risk factor for CVD (42). While one sex difference has actually been proposed for CRP plasma levels (43, 44), in our study we only found that this sex difference was due mainly to the diab group, as no sex differences were observed in the non-diab or new-diab groups.

Over the last few years, the association between plasma levels of TMAO and an increased risk of CVD has been highlighted. Several mechanisms have been proposed, including the inhibition of cholesterol transportation, the induction of foam cell formation and platelet reactivity (45, 46). Moreover, serum TMAO levels are associated with intima-media thickness, considered an early marker of atherosclerosis (32), and more recently an indicator of arterial injury (47, 48). Our study showed that the plasma levels of TMAO were lower in CHD non-diab women than CHD non-diab men, whereas no sex differences were observed in the new-diab group and diab groups. Moreover, IMT-CC and the number of plaques were lower in CHD non-diab women than in CHD non-diab men, whereas no sex differences were observed in the new-diab group and diab groups. Moreover, it has been described that plaque erosion is more prevalent in women whereas prevalence of plaque rupture and vulnerability increased with age in women but not in men (8). In addition, it has been also described a higher prevalence of rupture in women after menopause (49). Taken together, these results suggest that the cardiovascular risk associated to T2DM in CHD women is higher than non-T2DM CHD women, whereas it does change in CHD T2DM men as compared with non-T2DM CHD men. Of note, the analysing of IMT-CC and the carotid plaques in CHD patients according to the sex and the diabetic status has not been previously reported, to the best of our knowledge.

The CVD is influenced by sex, and men have generally higher prevalence than women (2), although once women have developed CVD, they have a higher risk of cardiovascular mortality than men (3). In line with this, we have previously shown that overall, CHD men had higher TMAO plasma levels than CHD women, together with higher IMT-CC, a larger number of carotid plaques and lower cholesterol efflux than CHD women, which may partially account for the sex dimorphism in the prevalence of CVD (33). The current work showed these differences when comparing CHD non-diab men and CHD non-diab women, whereas no differences were found between CHD diab men and CHD diab women, suggesting that the potential cardioprotection observed in women against CVD development, compared with men, is reduced by the presence of T2DM. In fact, the co-occurrence of CHD with T2DM markedly increases the risk of macrovascular complication and mortality (21, 22). Based on our results, we suggest that the condition of T2DM may at least partially cancel out any sex differences in endotoxemia and TMAO plasma levels in CHD patients, therefore influencing CVD development. Thus, although the contribution of T2DM to the cardiovascular risk has been previously described, taken together, our results point to a higher cardiovascular risk associated to T2DM in women than in men. In fact, we explored the first time the sex differences in the added cardiovascular risk associated to the co-occurrence of CHD with T2DM.

Our results showed that the presence of T2DM cancels out the reduced endotoxemia and TMAO levels observed in non-diabetic CHD women, compared with CHD men. Presumably, this may account for the higher percentage of CHD patients with T2DM in women than in men. This idea is also supported by the fact that the prevalence in the CORDIOPREV population of MetS, which has not previously been described for CHD patients. Our study showed that the prevalence of MetS a condition with elevated cardiovascular risk (12), was higher in CHD women than in CHD men. Moreover, the incidence of MetS in women was higher than in men. In addition, MetS is associated to a higher risk of T2DM development (50).

Our study has the limitations that the contribution of T2DM to the cardiovascular risk according to the sex was not the primary outcome of CORDIOPREV study but it allowed us to associate, at least observationally, the sex differences in endotoxemia and TMAO plasma levels with cardiovascular risk according to T2DM status in CHD patients. Further studies are need to fully understand this relationship.

Our results suggest the sex differences in CHD patients are influenced by the presence of T2DM. Overall, sex differences in endotoxemia and TMAO plasma levels were less in patients who had CHD and T2DM simultaneously. However, despite similar endotoxemia between sexes in CHD T2DM patients was observed, CHD T2DM women had higher levels of inflammation than CHD T2DM men. Taken together, our results also suggest that the cardiovascular risk associated to T2DM is higher in women than in men.

## Data Availability

The datasets presented in this article are not readily available because collaborations with the CORDIOPREV Study are open to Biomedical Institutions, always after an accepted proposal for scientific work. Depending on the nature of the collaboration, electronic data, hard copy data, or biological samples should be provided. All collaborations will be made after a collaboration agreement. Terms of the collaboration agreement will be specific for each collaboration, and the extent of the shared documentation (i.e., deidentified participant data, data dictionary, biological samples, hard copy, or other specified data sets) will be also specifically established in the light of each work. Requests to access the datasets should be directed to the corresponding author, antonio.camargo@imibic.org.

## Supplementary material

Supplementary Figure S1 Relationship of the anthropometric and biochemical variables with T2DM by multiple logistic regression analysis. T2DM, type 2 diabetes mellitus. The analysis was carried out including the baseline values for age, body mass index (BMI), waist circumference, insulin, total cholesterol (TC), high-density lipoprotein cholesterol (HDL-c), low-density lipoprotein cholesterol (LDL-c), triglycerides (TAG), C-reactive protein (CRP), and blood pressure (BP) as dependent variables and type 2 diabetes mellitus as independent variable (new-diab and diab groups together vs non-diab group). CHD, coronary heart disease.

Supplementary Figure S2 Baseline LPS, LBP and TMAO plasma levels and changes after 3 years of the consumption of the low-fat diet according to sex in each of the T2DM group. LPS, plasma levels of lipopolysaccharide; LBP, plasma levels of lipopolysaccharide binding protein; TMAO, plasma levels of trimethylamine N-oxide; T2DM, type 2 diabetes mellitus; CHD, coronary heart disease; y0, baseline; y3, after 3 years of the consumption of the low-fat diet; non-diab, CHD patients without type 2 diabetes mellitus; new-diab, CHD patients with recently diagnosed type 2 diabetes mellitus, and without treatment for diabetes; diab, CHD patients with type 2 diabetes mellitus, and under treatment for diabetes. ANOVA for repeated measures. P(sex), *p*-value according to the sex; P(time), *p*-value for time; P(inter), *p*-value for interaction between sex and time.

Supplementary Figure S3 Baseline LPS, LBP and TMAO plasma levels and changes after 3 years of the consumption of the Mediterranean diet according to sex in each of the T2DM group. LPS, plasma levels of lipopolysaccharide; LBP, plasma levels of lipopolysaccharide binding protein; TMAO, plasma levels of trimethylamine N-oxide; T2DM, type 2 diabetes mellitus; CHD, coronary heart disease; y0, baseline; y3, after 3 years of the consumption of the Mediterranean diet; non-diab, CHD patients without type 2 diabetes mellitus; new-diab, CHD patients with recently diagnosed type 2 diabetes mellitus, and without treatment for diabetes; diab, CHD patients with type 2 diabetes mellitus, and under treatment for diabetes. ANOVA for repeated measures. P(sex), *p*-value according to the sex; P(time), *p*-value for time; P(inter), *p*-value for interaction between sex and time.

Supplementary Figure S4 Baseline LPS, LBP and TMAO plasma levels and changes after 3 years in men according to the diet in each of the T2DM group. LPS, plasma levels of lipopolysaccharide; LBP, plasma levels of lipopolysaccharide binding protein; TMAO, plasma levels of trimethylamine N-oxide; T2DM, type 2 diabetes mellitus; CHD, coronary heart disease; y0, baseline; y3, after 3 years of the consumption of the Mediterranean diet; non-diab, CHD patients without type 2 diabetes mellitus; new-diab, CHD patients with recently diagnosed type 2 diabetes mellitus, and without treatment for diabetes; diab, CHD patients with type 2 diabetes mellitus, and under treatment for diabetes. ANOVA for repeated measures. P(diet), *p*-value according to the sex; P(time), *p*-value for time; P(inter), *p*-value for interaction between diet and time.

Supplementary Figure S5 Baseline LPS, LBP and TMAO plasma levels and changes after 3 years in women according to the diet in each of the T2DM group. LPS, plasma levels of lipopolysaccharide; LBP, plasma levels of lipopolysaccharide binding protein; TMAO, plasma levels of trimethylamine N-oxide; T2DM, type 2 diabetes mellitus; CHD, coronary heart disease; y0, baseline; y3, after 3 years of the consumption of the Mediterranean diet; non-diab, CHD patients without type 2 diabetes mellitus; new-diab, CHD patients with recently diagnosed type 2 diabetes mellitus, and without treatment for diabetes; diab, CHD patients with type 2 diabetes mellitus, and under treatment for diabetes. ANOVA for repeated measures. P(diet), *p*-value according to the sex; P(time), *p*-value for time; P(inter), *p*-value for interaction between diet and time.
