# Supplementary material for: Sex-differences in endotoxemia and trimethylamine N-oxide according to the diet and type 2 diabetes status in coronary heart disease patients: from the CORDIOPREV study
Source: Front Cardiovasc Med. 2025 Oct 21;12:1527406. doi: 10.3389/fcvm.2025.1527406 (PMC12582939; doi:10.3389/fcvm.2025.1527406)
Supplement: Supplementary file 1 [file Table1.docx]

Supplementary Material

| Analyte | Molecular formula | Retention time (min) | Quantitation transition (*m/z*) | Q1 voltage (V) | Collision energy (eV) |
| --- | --- | --- | --- | --- | --- |
| TMAO | C_3_H_9_NO | 0.95 | 76.1 → 58.1 | 60 | 22 |
| TMAO-d_9_ | C_3_D_9_NO | 0.96 | 85.2 → 66.1 | 40 | 26 |

**Supplementary Table S1.** Multiple reaction monitoring parameters for determination of TMA and TMAO by LC–MS/MS.
