# Supplementary material for: Sex-differences in endotoxemia and trimethylamine N-oxide according to the diet and type 2 diabetes status in coronary heart disease patients: from the CORDIOPREV study
Source: Front Cardiovasc Med. 2025 Oct 21;12:1527406. doi: 10.3389/fcvm.2025.1527406 (PMC12582939; doi:10.3389/fcvm.2025.1527406)
Supplement: Supplementary file 2 [file Table2.docx]

Supplementary Material

|  | *non-MetS* | | *MetS* | | *p-value* | |
| --- | --- | --- | --- | --- | --- | --- |
| Men (%) | 43.77 | | 56.23 | | 0.010 | |
| Women (%) | 33.14 | | 66.86 | |  | |
|  | *non-AHT* | | *AHT* | | *p-value* | |
| Men (%) | 31.68 | | 67.84 | | 0.583 | |
| Women (%) | 29.71 | | 70.29 | |  | |
|  | *TAG* | | *high-TAG* | | *p-value* | |
| Men (%) | 68.08 | | 31.80 | | 0.688 | |
| Women (%) | 69.71 | | 30.29 | |  | |
|  | *Glucose* | | *high-glucose* | | *p-value* | |
| Men (%) | 43.17 | | 56.71 | | 0.743 | |
| Women (%) | 44.57 | | 55.43 | |  | |
|  | *WC* | | *high-WC* | | *p-value* | |
| Men (%) | 34.34 | | 65.42 | | <0.001 | |
| Women (%) | 18.29 | | 81.71 | |  | |
|  | *HDL* | | *low-HDL* | | *p-value* | |
| Men (%) | 47.52 | | 52.24 | | 0.001 | |
| Women (%) | 34.29 | | 65.71 | |  | |
|  | |  | |  | |  |
|  | | *Men (%)* | | *Women (%)* | | *p-value* |
| *0 MetS criteria* | | 3.15 | | 1.17 | | 0.037 |
| *1 MetS criteria* | | 14.77 | | 14.86 | |  |
| *2 MetS criteria* | | 25.79 | | 17.14 | |  |
| *3 MetS criteria* | | 25.06 | | 26.29 | |  |
| *4 MetS criteria* | | 23.49 | | 26.29 | |  |
| *5 MetS criteria* | | 7.75 | | 13.71 | |  |

**Supplementary Table S2.** **Sex-dependent prevalence of MetS in CHD patients.** MetS: metabolic syndrome. CHD: coronary heart disease. MetS criteria were defined according to the Third Report of the National Cholesterol Education Program (NCEP) Expert Panel on Detection, Evaluation and Treatment of High Blood Cholesterol in Adults (Adult Treatment Panel III) to assess the presence of MetS (Expert Panel on Detection and Treatment of High Blood Cholesterol in, 2001). AHT: arterial hypertension. TAG: triacylglycerides. WC: Waist circumference. HDL: high-density lipoprotein cholesterol. The chi-square test was applied to establish differences in MetS prevalence and MetS criteria analysis.
