# Supplementary figures and images for: Sex-differences in endotoxemia and trimethylamine N-oxide according to the diet and type 2 diabetes status in coronary heart disease patients: from the CORDIOPREV study
Source: Front Cardiovasc Med. 2025 Oct 21;12:1527406. doi: 10.3389/fcvm.2025.1527406 (PMC12582939; doi:10.3389/fcvm.2025.1527406)

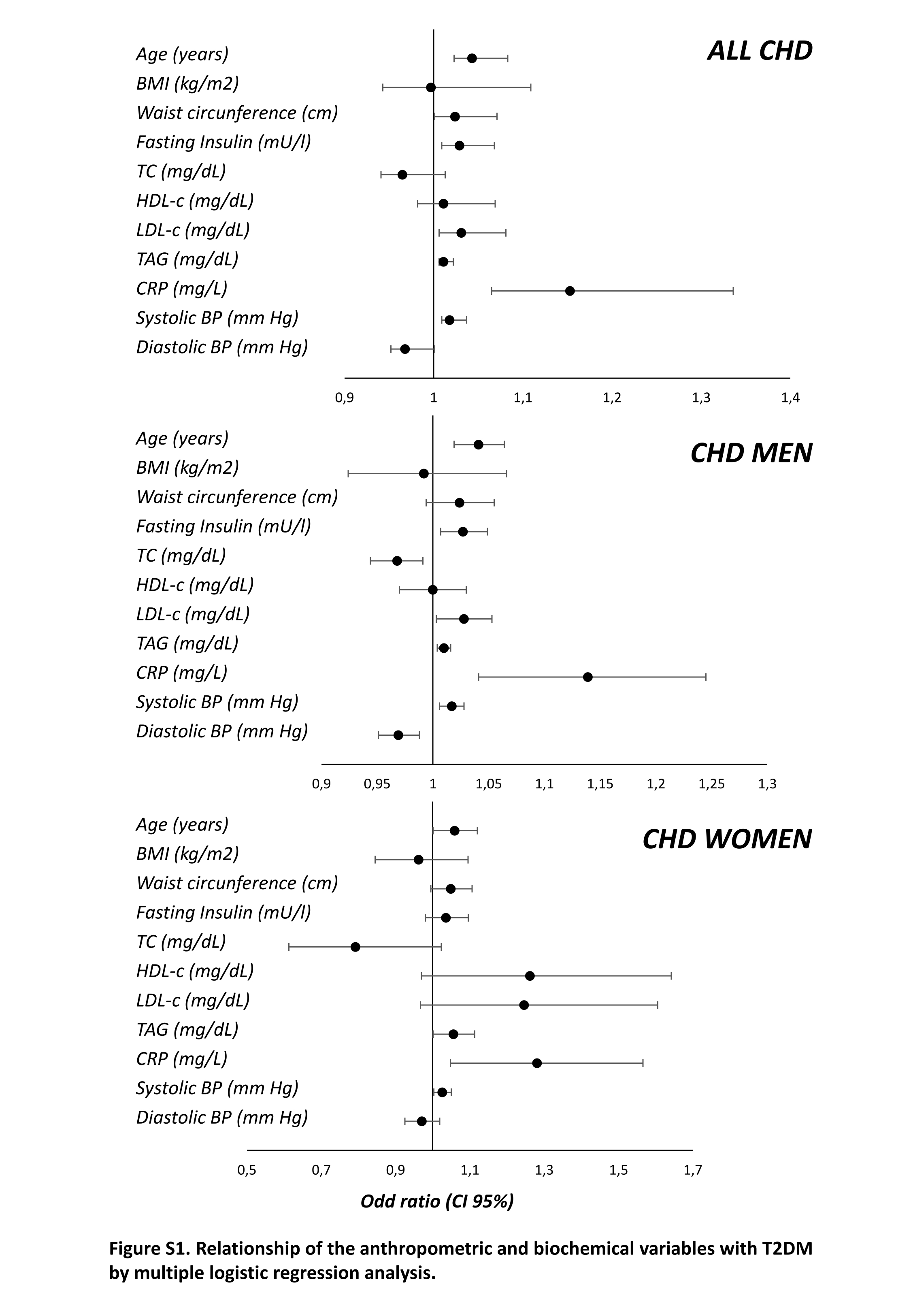

Supplement: Supplementary Figure S1 — Relationship of the anthropometric and biochemical variables with T2DM by multiple logistic regression analysis. T2DM, type 2 diabetes mellitus. The analysis was carried out including the baseline values for age, body mass index (BMI), waist circumference, insulin, total cholesterol (TC), high-density lipoprotein cholesterol (HDL-c), low-density lipoprotein cholesterol (LDL-c), triglycerides (TAG), C-reactive protein (CRP), and blood pressure (BP) as dependent variables and type 2 diabetes mellitus as independent variable (new-diab and diab groups together vs non-diab group). CHD, coronary heart disease. [file Image1.jpeg]

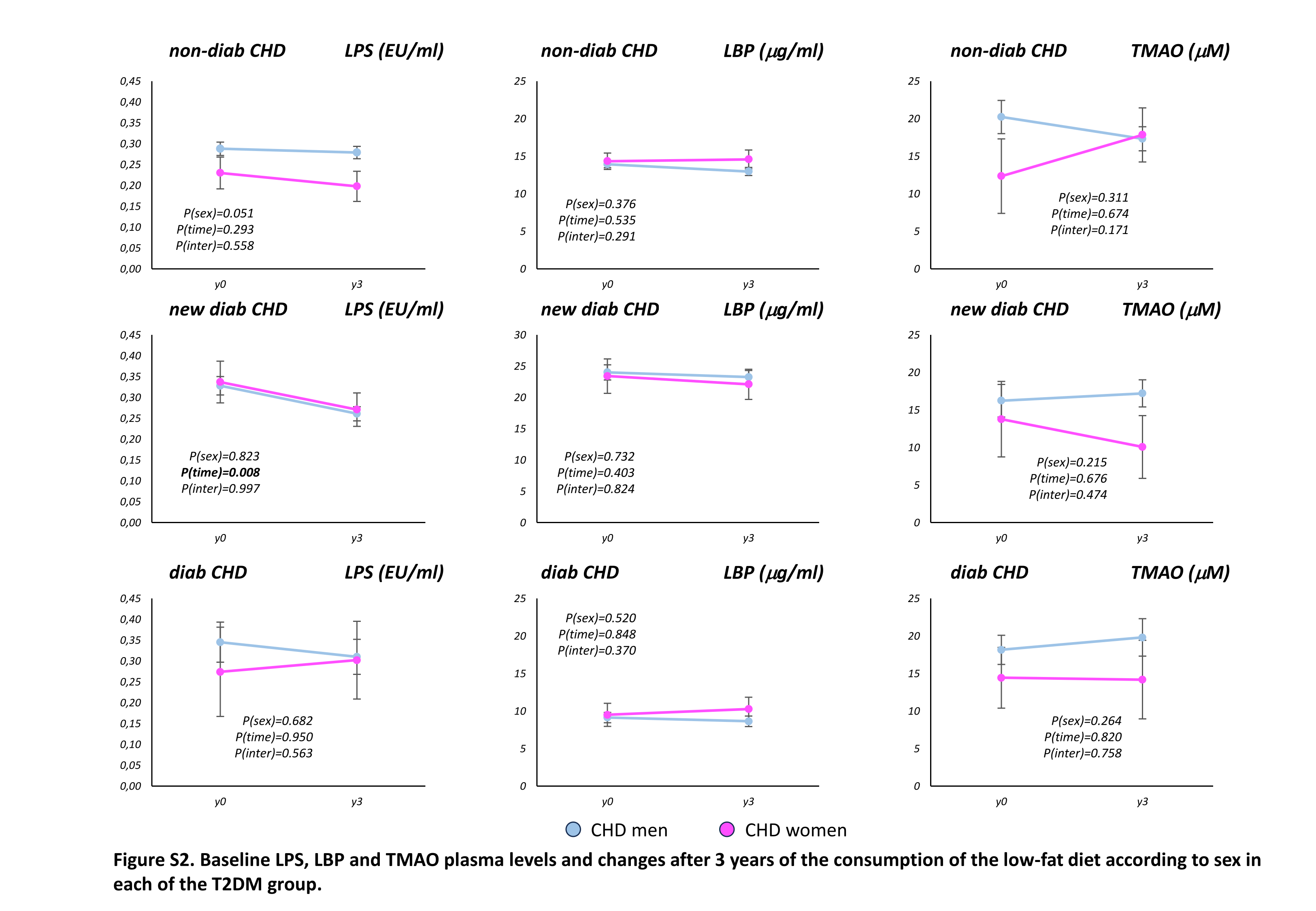

Supplement: Supplementary Figure S2 — Baseline LPS, LBP and TMAO plasma levels and changes after 3 years of the consumption of the low-fat diet according to sex in each of the T2DM group. LPS, plasma levels of lipopolysaccharide; LBP, plasma levels of lipopolysaccharide binding protein; TMAO, plasma levels of trimethylamine N-oxide; T2DM, type 2 diabetes mellitus; CHD, coronary heart disease; y0, baseline; y3, after 3 years of the consumption of the low-fat diet; non-diab, CHD patients without type 2 diabetes mellitus; new-diab, CHD patients with recently diagnosed type 2 diabetes mellitus, and without treatment for diabetes; diab, CHD patients with type 2 diabetes mellitus, and under treatment for diabetes. ANOVA for repeated measures. P(sex), p-value according to the sex; P(time), p-value for time; P(inter), p-value for interaction between sex and time. [file Image2.jpeg]

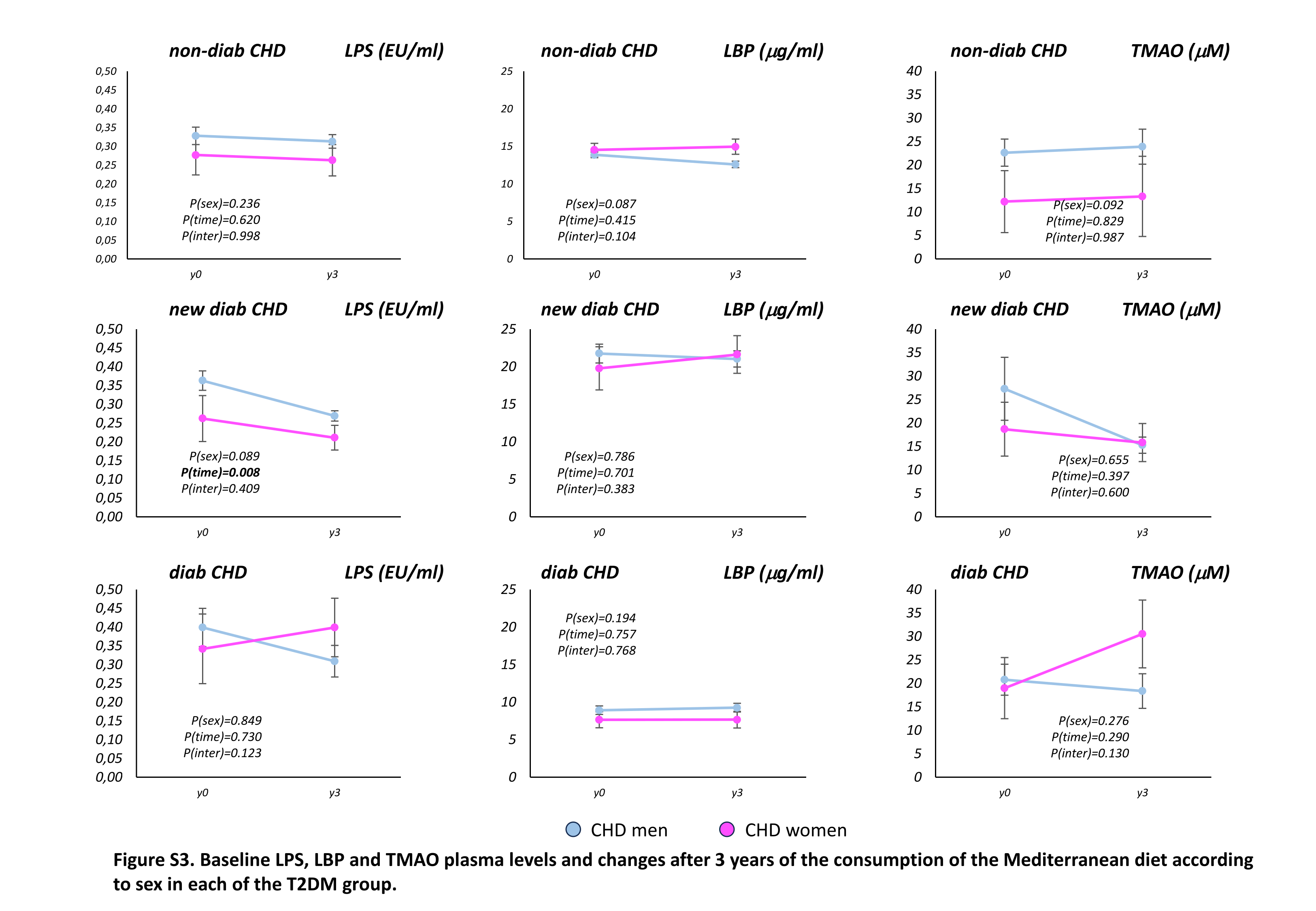

Supplement: Supplementary Figure S3 — Baseline LPS, LBP and TMAO plasma levels and changes after 3 years of the consumption of the Mediterranean diet according to sex in each of the T2DM group. LPS, plasma levels of lipopolysaccharide; LBP, plasma levels of lipopolysaccharide binding protein; TMAO, plasma levels of trimethylamine N-oxide; T2DM, type 2 diabetes mellitus; CHD, coronary heart disease; y0, baseline; y3, after 3 years of the consumption of the Mediterranean diet; non-diab, CHD patients without type 2 diabetes mellitus; new-diab, CHD patients with recently diagnosed type 2 diabetes mellitus, and without treatment for diabetes; diab, CHD patients with type 2 diabetes mellitus, and under treatment for diabetes. ANOVA for repeated measures. P(sex), p-value according to the sex; P(time), p-value for time; P(inter), p-value for interaction between sex and time. [file Image3.jpeg]

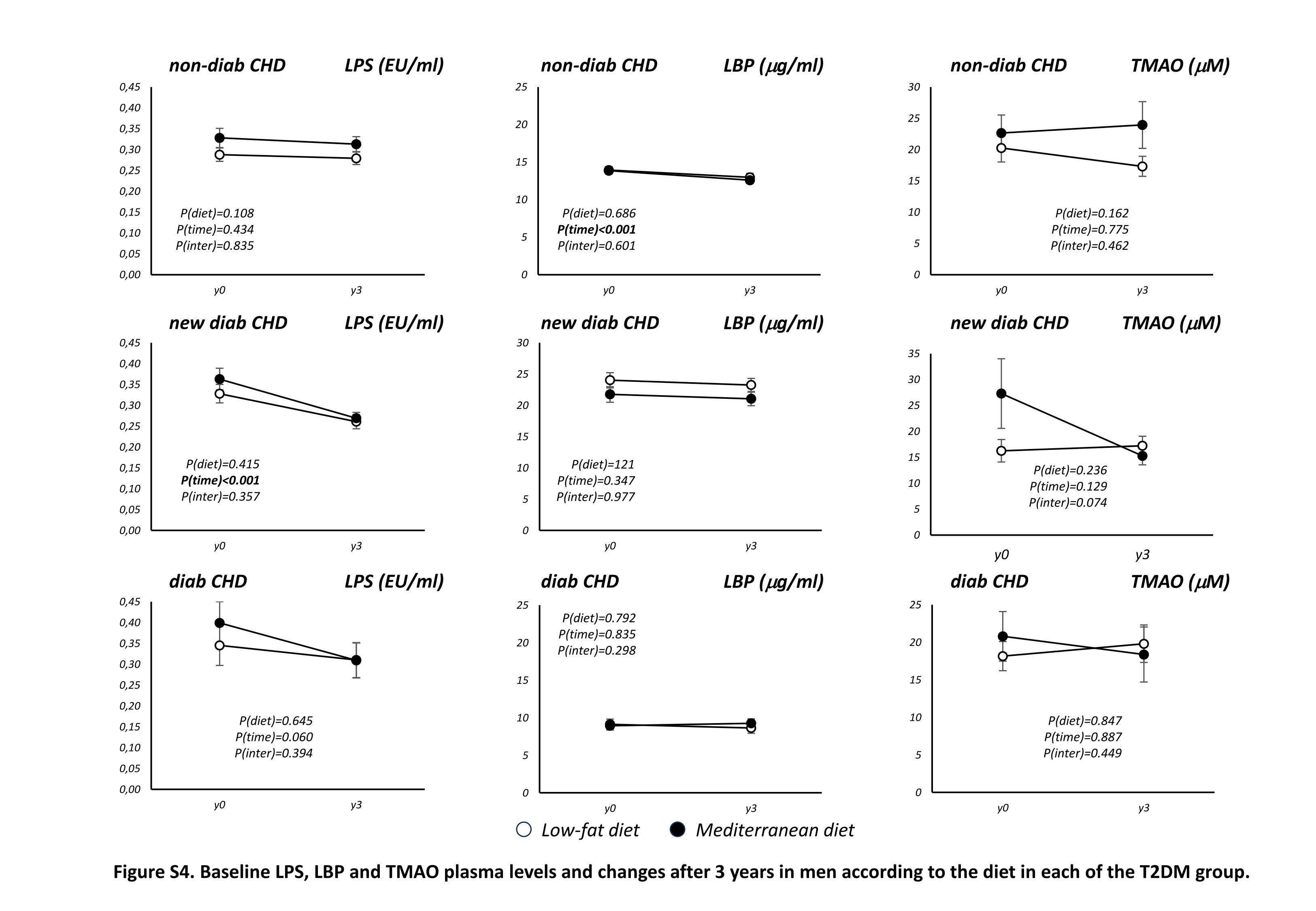

Supplement: Supplementary Figure S4 — Baseline LPS, LBP and TMAO plasma levels and changes after 3 years in men according to the diet in each of the T2DM group. LPS, plasma levels of lipopolysaccharide; LBP, plasma levels of lipopolysaccharide binding protein; TMAO, plasma levels of trimethylamine N-oxide; T2DM, type 2 diabetes mellitus; CHD, coronary heart disease; y0, baseline; y3, after 3 years of the consumption of the Mediterranean diet; non-diab, CHD patients without type 2 diabetes mellitus; new-diab, CHD patients with recently diagnosed type 2 diabetes mellitus, and without treatment for diabetes; diab, CHD patients with type 2 diabetes mellitus, and under treatment for diabetes. ANOVA for repeated measures. P(diet), p-value according to the sex; P(time), p-value for time; P(inter), p-value for interaction between diet and time. [file Image4.jpeg]

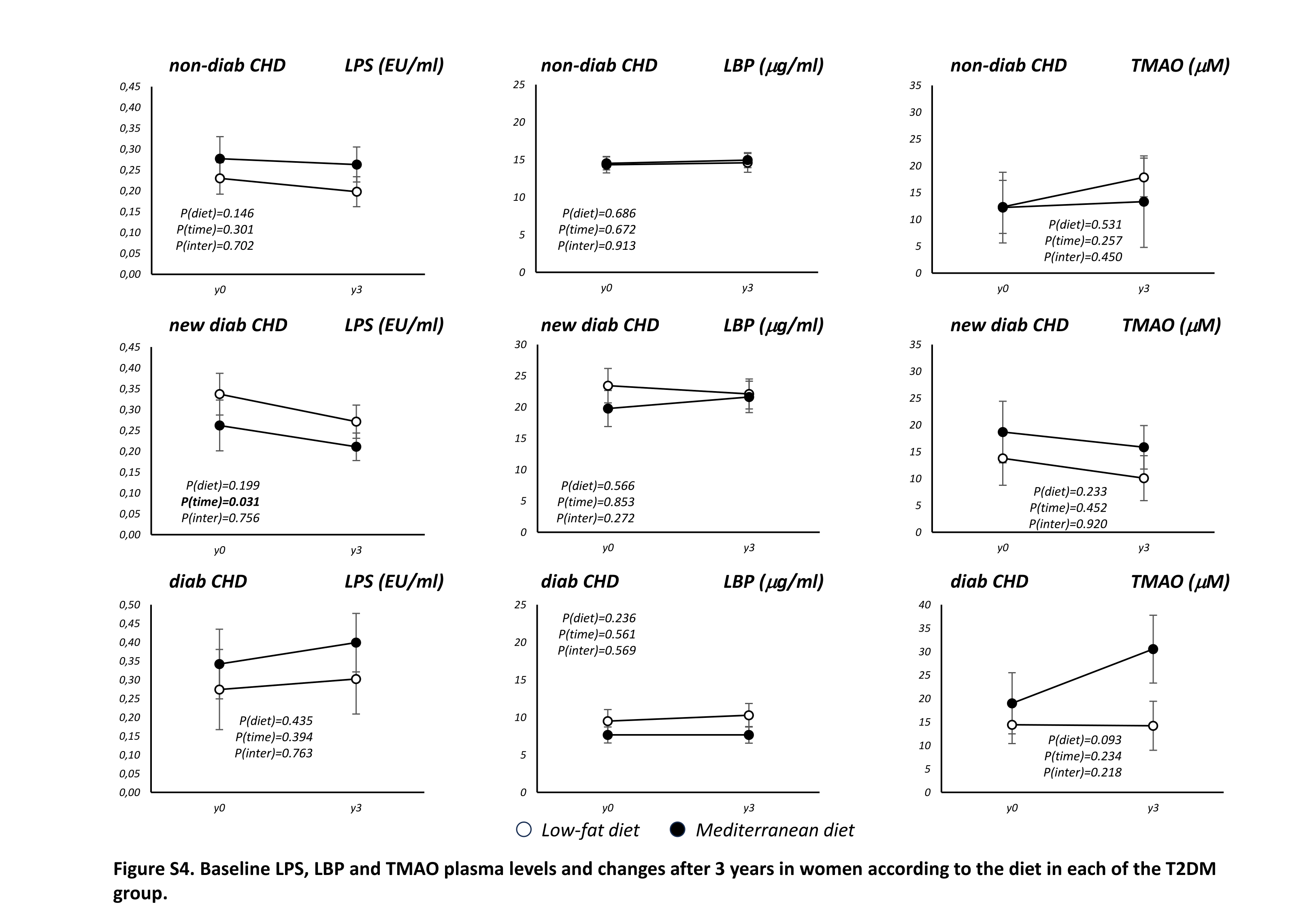

Supplement: Supplementary Figure S5 — Baseline LPS, LBP and TMAO plasma levels and changes after 3 years in women according to the diet in each of the T2DM group. LPS, plasma levels of lipopolysaccharide; LBP, plasma levels of lipopolysaccharide binding protein; TMAO, plasma levels of trimethylamine N-oxide; T2DM, type 2 diabetes mellitus; CHD, coronary heart disease; y0, baseline; y3, after 3 years of the consumption of the Mediterranean diet; non-diab, CHD patients without type 2 diabetes mellitus; new-diab, CHD patients with recently diagnosed type 2 diabetes mellitus, and without treatment for diabetes; diab, CHD patients with type 2 diabetes mellitus, and under treatment for diabetes. ANOVA for repeated measures. P(diet), p-value according to the sex; P(time), p-value for time; P(inter), p-value for interaction between diet and time. [file Image5.jpeg]
